# Polyindole Embedded Nickel/Zinc Oxide Nanocomposites for High-Performance Energy Storage Applications

**DOI:** 10.3390/nano13030618

**Published:** 2023-02-03

**Authors:** Huriya Humayun, Bushra Begum, Salma Bilal, Anwar ul Haq Ali Shah, Philipp Röse

**Affiliations:** 1 National Centre of Excellence in Physical Chemistry 1, University of Peshawar, Peshawar 25120, Pakistan; 2 Institute of Chemical Science, University of Peshawar, Peshawar 25120, Pakistan; 3 Institute for Applied Materials—Electrochemical Technologies, Karlsruhe Institute of Technology (KIT), 76131 Karlsruhe, Germany

**Keywords:** supercapacitors, ternary hybrid electrodes, conductive polymers, oxidative polymerization, one-pot synthesis

## Abstract

Conducting polymers integrated with metal oxides create opportunities for hybrid capacitive electrodes. In this work, we report a one-pot oxidative polymerization for the synthesis of integrated conductive polyindole/nickel oxide (PIn/NiO), polyindole/zinc oxide (PIn/ZnO), and polyindole/nickel oxide/zinc oxide (PNZ). The polymers were analyzed thoroughly for their composition and physical as well as chemical properties by X-ray diffraction (XRD), scanning electron microscopy (SEM), Fourier transform infrared spectroscopy (FTIR), ultraviolet–visible spectroscopy (UV–Vis), and thermogravimetric analysis (TGA). The PIn and its composites were processed into electrodes, and their use in symmetrical supercapacitors in two- and three-electrode setups was evaluated by cyclic voltammetry (CV), galvanostatic discharge (GCD), and electrochemical impedance spectroscopy (EIS). The best electrochemical charge storage capability was found for the ternary PNZ composite. The high performance directly correlates with its uniformly shaped nanofibrous structure and high crystallinity. For instance, the symmetrical supercapacitor fabricated with PNZ hybrid electrodes shows a high specific capacitance of 310.9 F g^−1^ at 0.5 A g^−1^ with an energy density of 42.1 Wh kg^−1^, a power density of 13.2 kW kg^−1^, and a good cycling stability of 78.5% after 5000 cycles. This report presents new electrode materials for advanced supercapacitor technology based on these results.

## 1. Introduction

Green electrical energy production and storage are of great importance for mitigating environmental issues, and researchers are intensively searching for new ways to produce renewable energy [1,2,3,4]. They are becoming promoters of developing new efficient and sustainable energy storage systems, especially for high energy storage capacity at moderate power density [5]. Supercapacitors are often integrated into existing energy storage systems to meet these requirements. They offer significant advantages over batteries in terms of fast charge/discharge characteristics, lower cost, and low carbon footprint. Additionally, supercapacitors have a far longer life cycle. Therefore, they are utilized in numerous advanced technologies with high energy demands, such as hybrid vehicles, uninterruptible power supplies, and electronics [4]. Faradaic supercapacitors (FS) typically use transition metal oxides such as manganese oxide (MnO_2_), nickel oxide (NiO), and zinc oxide (ZnO). They are often combined with conductive polymers such as polypyrrole (PPy) and polyaniline (PANI) for performance improvement. They have high specific capacitance and good charge/discharge characteristics, as they store charge via redox reactions at the electrode–electrolyte interface. However, there is still a need to improve their cycling stability and electrochemical as well as mechanical stability [6]. PANI has been leading the field as a pseudocapacitive polymeric electrode material with its high temperature stable, optical, electrical, and electrochemical properties for a decade [7]. In addition, PANI composites with metal oxides have been attractive candidates in supercapacitors, photocatalytic degradation, and many other applications [8]. However, advancement is becoming slow. As a result, attention turned to other conductive polymers, such as polyindoles (PIn), with good reversible redox activity, high thermal and electrical stability, and low synthesis cost. Improvements are sought in all aspects, including structural stability, ion transport optimization in the porous structures, and enhancement of electrical conductivity. All this contributes to the improvement of the capacitive properties. An interesting approach is the fabrication of PIn hybrid electrodes in combination with transition metal oxides. As with PANI and PPy, an improvement in capacitance is expected [9].

A variety of PIn-based composites have been reported in the literature. For example, Zhou et al. synthesized ternary PIn-integrated carbon nanotubes and cobalt oxide (CNTs/PIn/Co_3_O_4_) as hybrid electrode materials and achieved a specific capacitance of 443.5 F g^−1^ at 1 A g^−1^ [10]. The energy and power densities were 42.9 Wh kg^−1^ and 0.4 kW kg^−1^, respectively, and 90% of the capacitance was retained after 5000 cycles. Similarly, a ternary composite electrode of PIn-reduced graphene oxide and titanium dioxide (PIn-rGO-TiO_2_) was reported by Ram et al., which exhibited a specific capacitance of 482.9 F g^−1^ and a 114% capacitance retention (referring to the specific capacitance of the first cycle measured) at 1 A g^−1^ [11]. Majumder et al. reported a ternary composite of PIn, carbon black, and molybdenum disulfide (PIN/CB/MoS_2_), which exhibited a specific capacitance of 442 F g^−1^ and charged retention of 92% (after 5000 cycles) [12]. In addition, Zhou et al. reported a vanadium pentoxide/PIn decorated activated carbon cloth electrode (V_2_O_5_/PIn@AC) with a specific capacitance of 535.5 F g^−1^, an energy density of 38.7 Wh kg^−1^, a power density of 900 W kg^−1^, and a capacity retention of 91% after 5000 cycles [13].

The reports show that developing hybrid electrodes consisting of multiple components is a beneficial and plausible approach for improving capacitive performance. However, the ternary hybrid electrodes are expensive due to the high-cost carbonaceous materials and the chosen metal oxides. The challenges for further improvements include selecting suitable components for a high-performance hybrid electrode, reducing toxic and expensive metal oxides, and ensuring long lifetime. So far, no FS system has met all the requirements to achieve good sustainability and energy efficiency.

For PANI and PPy, the integration of low-cost metal oxides such as NiO and zinc oxide (ZnO) has shown promising results. In this context, integrating those two metal oxides into PIn at the same time may also result in the properties’ synergism, leading to hybrid electrodes with improved capacitive properties. NiO and ZnO have high theoretical specific capacitance, unusual redox activity, and are low-cost and toxic. Their metal oxides are known to improve the specific surface area and electrical properties by changing the morphology and structural properties compared to bare polyindole [14,15,16,17,18].

In the current study, we report on novel NiO and ZnO doped polyindole composites synthesized by a simple and low-cost chemical oxidative one-pot procedure. In addition, the composites were systematically investigated for their structural, physicochemical, and electrochemical properties in FS.

## 2. Materials and Methods

### 2.1. Chemicals

The following chemicals were used for the synthesis: Indole (C8H7N, >99%), nickel oxide (>99.9%), zinc oxide (>99%), ammonium persulfate (APS, >98%), dodecylbenzene sulfonic acid (DBSA, >95%), acetone (99%), sulfuric acid (98%), and chloroform (>99.8%) were purchased from Sigma-Aldrich (St. Louis, MI, USA). Isopropanol (99.5%) and toluene (99%) were purchased from Daejung (Seoul, Republic of Korea) and Scharlau (CAT, Barcelona, Spain), respectively. Deionized water (>18 MQ, Merck MilliQ, Burlington, MA, USA) was used for all aqueous solution applications.

### 2.2. One-Pot Synthesis of the Polyindole/Metal Oxide Composites

**Scheme 1:**
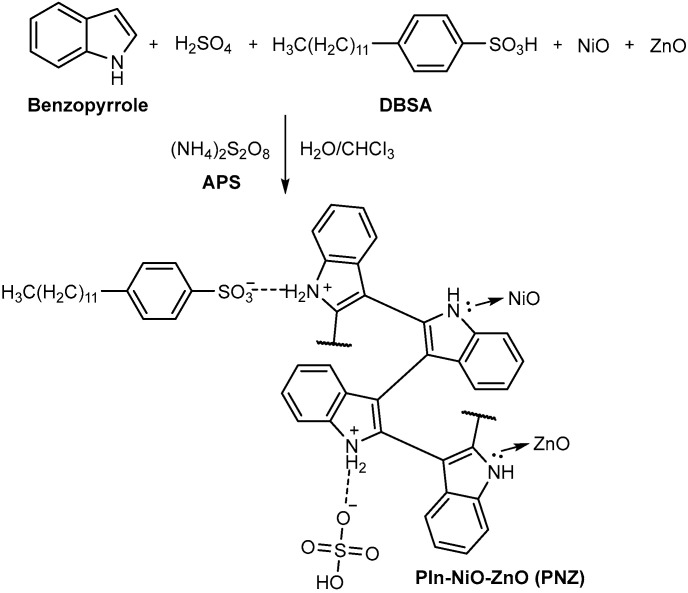
Synthesis of PIn-NiO-ZnO (PNZ-1 to -4) via one-pot oxidative polymerization.

H_2_SO_4_ (100 mL, 1.0 M) and DBSA (1.0 mL, 2.62 mmol, 1.0 eq) were added to a 250 mL round-bottom flask. Subsequently, APS (1.6 g, 7.01 mmol, 2.7 eq), NiO (0.21 g, 2.62 mmol, 1.0 eq), and ZnO (0.10 g (PNZ-1), 0.20 g (PNZ-2), 0.30 g (PNZ-3), 0.40 g (PNZ-4), 2.46–4.91 mmol, 1.0–1.9 eq) were added. Last, indole (0.3 g, 2.56 mmol, 1.0 eq) in chloroform (0.3 mL) was added dropwise and stirred at room temperature for 24 h (Scheme 1). After completion, the dark green polyindole composite was filtered and washed with acetone/dest. water (1:1 *v*/*v*). The composite was dried at 60 °C in a vacuum for 24 h and stored in an airtight vial afterward. Pure PIn (without NiO and ZnO), PIn/NiO, and PIn/ZnO were prepared according to the same procedure.

### 2.3. Material Characterisation

The structural properties of the PIn composites were investigated using a Fourier transform infrared (FTIR) spectrometer (Affinity-1S FT-IR, Shimadzu, Columbia, MD, USA) in the scanning range of 450–4000 cm^−1^ and a resolution of 2 cm^−1^. X-ray diffractometry (Bruker D8 Advance, Karlsruhe, Germany) was conducted in a 2θ range of 10°–80°. The copper Kα radiation of a 1.54 Å wavelength was applied at a scan rate of 0.05°/s at 40 kV and 35 mA. Furthermore, the morphology and elemental composition of prepared PIn samples were investigated using scanning electron microscopy (SEM, Supra-55VPFEGSEM, ZEISS, Oberkochen, DE) and energy-dispersive X-ray spectroscopy (EDX, Oxford Instrument, Abingdon, UK) analysis. The software Nano Measurer (v. 1.2.5, Fudan University, Shanghai, China) was used to determine the mean particle sizes. Thermal degradation analysis was examined with a thermogravimetric analyzer (TGA, Netsch, Selb, Bavaria, Germany). Each sample was subject to heat with a heating rate of 10 °C/minute under a nitrogen atmosphere. Moreover, the electrical conductivity of each polymer was analyzed in the form of a pellet using a four-probe resistivity tester (Bridge Technology Co. Ltd., Boise, ID, USA).

### 2.4. Electrochemical Characterization

Electrochemical studies were performed with a Reference 3000 potentiostat/galvanostat/ZRA (Gamry Instruments, Warminster, PA, USA). The redox properties of each PIn composite were studied in a three-electrode setup utilizing a polymer-coated gold sheet working electrode (1 cm^2^), a bare gold sheet counter electrode (1 cm^2^), and a calomel reference electrode (Hg/Hg_2_Cl_2_ in KCl_sat_ in H_2_O). The prepared PIn composites were dip-coated with a 1:2 mixture of 2-propanol:toluene on a polished gold sheet. As the electrolyte, 1.0 M aqueous H_2_SO_4_ solution was used.

Cyclic voltammetry (CV) was performed at a scan rate of 100 mV s^−1^ in various potential ranges (E_min_ = −0.3 V, E_max_ = 0.7, 0.8, 0.9, 1.0, and 1.1 V). Furthermore, scan rate variations (20 to 100 mV s^−1^) were made for assessing capacitance retentions. In addition, galvanostatic charge/discharge (GCD) experiments were done at different current densities (0.35, 0.5, 1.0, 1.5, 3.5, and 5.0 A g^−1^) to determine the gravimetric capacitance. The specific gravimetric capacitance (*C_S_*) of the active composites is calculated from the CV and GCD curves using Equations (1) and (2) [19,20]:

(1) $$C_{S}=\int \frac{Id\nu }{2m\cdot v\cdot \Delta V}$$

(2) $$C_{S}=\frac{I\cdot \Delta t}{\Delta V\cdot m}$$

where ʃ *Idν* is the integrated area of the baseline-corrected CV curve, *m* (g) is the active composite mass, *I* (A) is the current, *ν* (mV s^−1^) is the scan rate, and Δ*V* (V) is the IR-corrected potential range.

Electrochemical impedance spectroscopy (EIS) was used to determine the internal resistance, interfacial charge transfer process at the electrode/electrolyte interface, and ion diffusion. The excitation amplitude was 5 mV_rms_, and the frequency range was set between 10^5^ Hz and 50 mHz.

The symmetric PNZ capacitor was analyzed using two identically coated gold sheets stacked with a separator (Whatman GF/A) in between and soaked with 100 μL of 1.0 M aqueous H_2_SO_4_ electrolyte solution. CV and GCD experiments were conducted in the same manner as in the three-electrode setup. The specific capacitance was determined from the GCD plots according to Equation (3) [21,22]:

(3) $$C_{S}=\frac{4I\cdot \Delta t}{\Delta V\cdot m}$$

The energy density (*E*) (Wh kg^−1^) and power density (*P*) (W kg^−1^) were estimated from Equations (4) and (5), respectively [21].

(4) $$E=\frac{C_{S}\cdot \Delta V^{2}}{8}$$

(5) $$P=\frac{E}{\Delta t}$$

The coulombic efficiency *η* (%) is calculated by Equation (6):

(6) $$\eta =\frac{t_{d}}{t_{c}}\cdot 100\%$$

where *t_d_* and *t_c_* are the charge and discharge times.

## 3. Results and Discussion

### 3.1. Physico-Chemical Characterisation of the Polyindole Composites

The physical and chemical material properties of polyindole (PIn), polyindole–nickel oxide composite (PIn/NiO), polyindole–zinc oxide composite (PIn/ZnO), and polyindole–nickel oxide/zinc oxide composite (PNZ-1 to -4) are described in the following.

**Figure 1:**
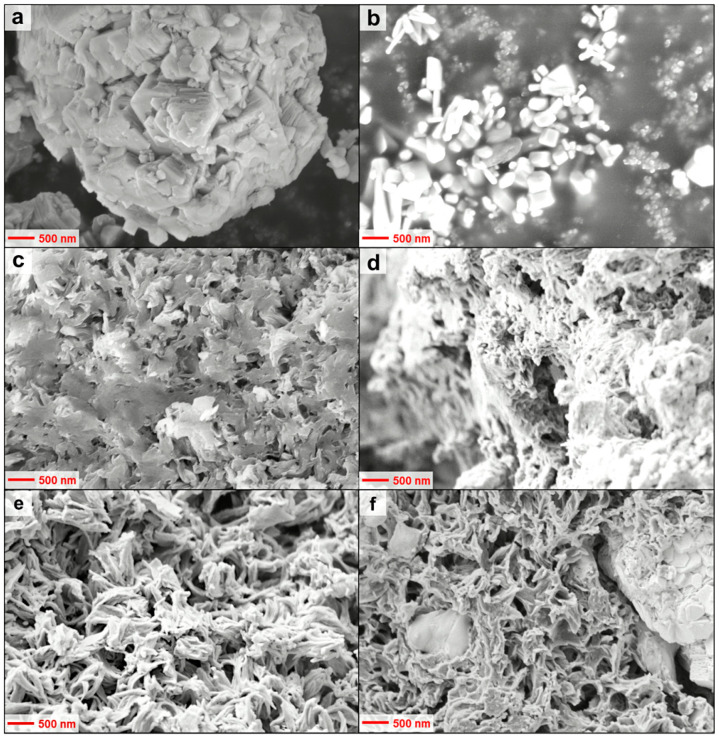
SEM-images of (**a**) NiO, (**b**) ZnO, (**c**) PIn, (**d**) PIn/NiO, (**e**) PIn/ZnO, (**f**) PNZ-3.

For supercapacitors, an electrode material’s structural characteristics and surface area are directly related to its capacitive properties. Therefore, a nano-structured morphology with the largest possible surface area and vacancies for good ion transport is crucial to achieving high electrochemical performance in a supercapacitor device. The SEM images of NiO, ZnO, and PIn, as well as the PIn metal oxide composites, are shown in Figure 1.

Nickel oxide used for the synthesis has a layered agglomerated structure of nano- and microparticles (Figure 1a) [23]. The flat particles are irregular and stacked on top of each other. They lead to a compact morphology. In contrast, the ZnO particles consist of various shapes, e.g., small rods and rectangles, which appear in clusters due to agglomeration [24]. The mean diameters of the individual NiO and ZnO particles were determined to be 32.6 nm and 73.7 nm, respectively, using image recognition software (Nano Measurer v1.25). From the SEM images shown in Figure 1c–f, it is evident that each polymer sample exhibited a heterogeneous and porous surface morphology. PIn possesses a coral-like flat structure (Figure 1c). With Pin/NiO, a much denser microstructure is obtained, which is made of fine nanosized particles with vacancies (ᴓ~100 nm) and has a rougher surface compared to pure PIn (Figure 1d). The average diameter of Pin/NiO nanoparticles was found to be 41.0 nm [22,24].

In contrast, uniform entangled nanostructures with a mean diameter of 38.7 nm occurred for Pin/ZnO (Figure 1e). The structure is highly porous, with many vacancies from 50–200 nm. The SEM image for PNZ-3 (0.3 g ZnO during synthesis) shows a similar structure with porous, intertwined nanostructures (Figure 1f). The mean diameter of the PNZ-3 nanostructure is about 46.2 nm, with vacancies of a mean diameter of 123.7 nm. Few larger clustered microparticles are integrated into the composite structure. Since the structures deviate strongly, it can be concluded that ZnO and NiO have been integrated into the PIn structure [22,25,26].

The structural features of all ternary PNZ hybrids are shown in Figure S1. The image of PNZ-1 (Figure S1a) shows agglomerated dense structures with very few vacancies. In PNZ-2, non-uniform nanostructures with particles on the surface were observed. A vast pore structure can be seen. PNZ-4 exhibits a heterogeneous agglomerated microstructure with non-uniform vacancies (Figure S1d). The change in surface morphology directly correlates with the ZnO concentration. The correlation indicates a strong intermolecular interaction between PIn and ZnO [27]. In contrast to the PNZ samples -1, -2, and -4, PNZ-3 exhibits the most uniform nanostructure. As a result, it can be deduced that PNZ-3 may serve as an excellent supercapacitor electrode material. Hence, pre-experiments could prove the much lower performance of PNZ-1, -2, and -4. Therefore, the following data for those samples are only shown in the SI.

The polymer matrix’s elemental composition of PIn, NiO, and ZnO was proven by EDX mapping analysis. Figures S2 and S3 show the EDX spectra and elemental mapping of PIn, PIn/NiO, PIn/ZnO, and the PNZ composites. For Pin, C, O, N, and S were found to be homogeneously distributed. The appearance of sulfur proves the incorporation of DBSA into the polymer structure [22]. For the composites, Ni and Zn were identified, respectively. For all samples, a homogenous distribution was observed with no visible agglomerates.

**Figure 2:**
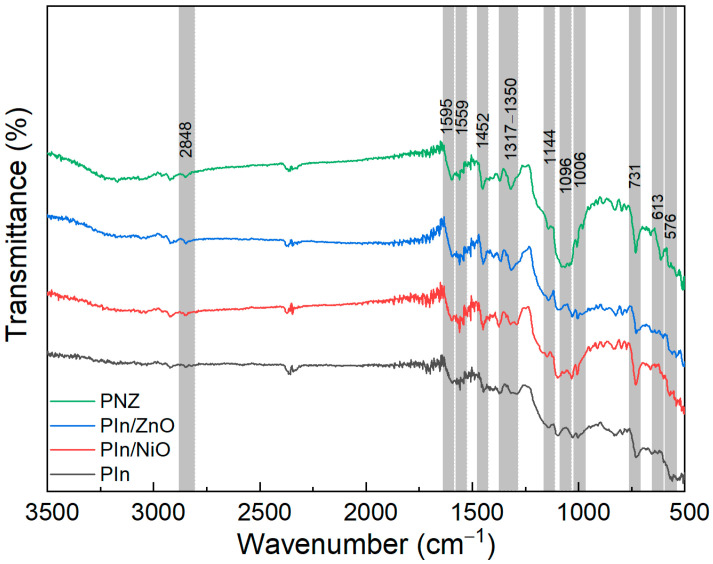
FTIR spectra of the synthesized PIn and its metal oxide composites.

FTIR and XRD further cross-checked the composition of the PIn and the composites. Figure 2 shows the FTIR spectra of PIn, PIn/NiO, PIn/ZnO, and PNZ-3.

Characteristic deformation vibrations for PIn are found at 2848 cm^−1^ (aromatic C-H), 1595 cm^−1^ (C-C), 1559 cm^−1^ (N-H), 1452 cm^−1^ and 1144 cm^−1^ (C-N), 1045 cm^−1^ (benzene ring), and 731 cm^−1^ (C-H). The pyrrole ring stretching vibration is at 1317–1350 cm^−1^. The features at 1006 cm^−1^ and 576 cm^−1^ are attributed to S=O and SO_3_^1−^ vibrations of DBSA and H_2_SO_4_. The incorporation of NiO and ZnO into the PIn structure is also observed; additional low-intensity bands at 613 cm^−1^ and 507 cm^−1^ for Ni-O and Zn-O, respectively, can be seen. Furthermore, the broadening of the N-H stretching vibration indicates the coordinative bonding of nitrogen to NiO and ZnO [22,24,28,29,30,31]. A slight change in peak position and intensity at 507 cm^−1^ was observed for the different ZnO concentrations. The FTIR spectra of the ternary NiO, ZnO, and PNZ composites are shown in Figure S4 [27].

**Figure 3:**
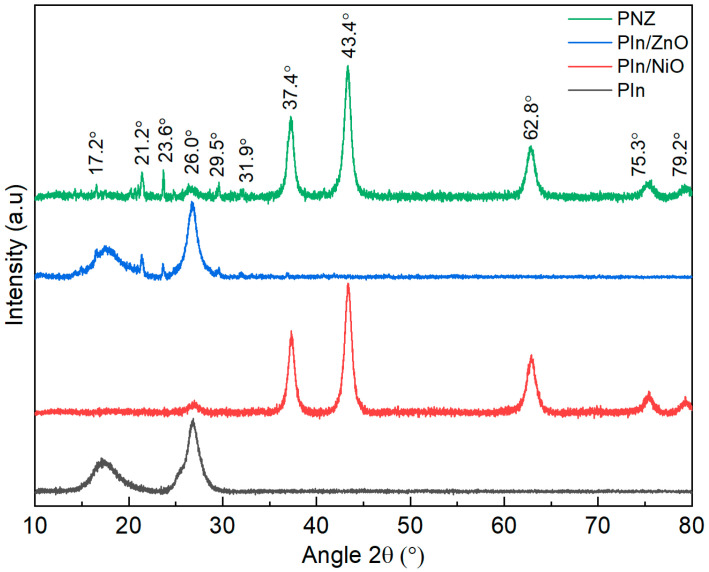
XRD spectra of the synthesized PIn and its metal oxide composites.

Additionally, XRD analysis was performed for all samples to validate further the incorporation of NiO and ZnO into the polymer structure and to identify whether the composites are amorphous or crystalline. The XRD patterns of the synthesized PIn, PIn/NiO, PIn/ZnO, and PNZ-3 are shown in Figure 3. The PIn shows low crystallinity, evident from the broad bands at 2θ = 17.2° and 26.0° [24]. The binary composites PIn/NiO and PIn/ZnO, as well as PNZ-3, possess a semi-crystalline structure. The PIn/NiO hybrid has a lower crystallinity than the other composites, evident from a broad band in the range of 2θ = 16–27°. In addition to the broad bands, the sharp reflection peaks at 2θ = 37.4°, 43.4°, 62.8°, 75.3°, and 79.2° were visible in the XRD spectrum of PIN/NiO, confirming the presence of face-centered crystalline NiO (JCPDS no. 04-0835) in the composite. The occurrence of these peaks is due to the incorporation of NiO into the polymer chain [22].

Similarly, PIn/ZnO showed characteristic broad bands (2θ = 17.2° and 26.0°) corresponding to low crystalline PIn and diffraction peaks at 2θ = 31.9°, 34.5°, 36.5°, 47.7°, 56.7°, 63.0°, 68.0°, and 69.2°, associated with the wurtzite structure of ZnO (JCPDS no. 36-1452). The decrease in intensities of these reflection lines reveals the low crystallinity associated with the ZnO. Unusual peaks were found at 2θ = 21.2°, 23.6°, and 29.5°. They are caused by the sterically hindering alkyl chain of the DBSA anions incorporated into the PIn backbone. The alkyl chain results in a disorder of the polymer structure and, thus, low crystallinity [23,28]. Moreover, the ternary PNZ-3 composite pattern exhibited all the diffraction peaks that could originate from the individual components (PIN, NiO, and ZnO) in the composite. Therefore, the appearance of broadened and less intense peaks at 2θ = 17.2° and 26.0° indicates the presence of a low crystallinity component of PIn. The prominent peaks at 2θ = 37.4°, 43.4°, 62.8°, and 75.3° indicate that the primary influence on the crystallinity of the polymer hybrid originates from NiO. The minor peaks at 2θ = 21.2°, 23.6°, and 29.5° correspond to ZnO in PNZ-3 [22,28]. Moreover, for PIn-NiO and PNZ, a decrease in the intensity of the PIn peaks is observed at 2θ = 17.2° and 26.0°. This trend indicates that the less crystalline PIn is subordinated in the presence of highly crystalline NiO. Thus, the XRD analysis indicates that both NiO and ZnO are integrated into the PIn matrix, thus confirming the successful synthesis of the PNZ-3 composite.

Additionally, the average particle size (*D*, nm) of the semi-crystalline composites was calculated using Scherrer’s Equation (6) [22]:

(7) $$D=K\cdot \lambda \cdot d\cdot \cos\theta^{-1}$$

where *θ* is the diffraction angle at peak position, *K* (0.9) is the Scherrer’s constant, *d* (rad) is the half-peak width, and *λ* (1.54 Å) is the X-ray wavelength. The mean particle sizes determined from the signature peaks (2*θ* = 21.2°, 23.6°, 29.5°, 37.4°, 43.4°, 62.8°, 75.3°) of ZnO and NiO in both binary and ternary composites peaks were 13.0 nm (PIn/NiO), 30.4 nm (PIn/ZnO), and 22.7 nm (PNZ-3). The calculated particle size from XRD and Scherrer’s formula is the primary particle size and lower than the average diameter measured by SEM. On the other hand, the particle size estimated from SEM is the secondary or aggregated particle size, which may relate to the actual performance (cf. 3.2. and 3.3.)

**Figure 4:**
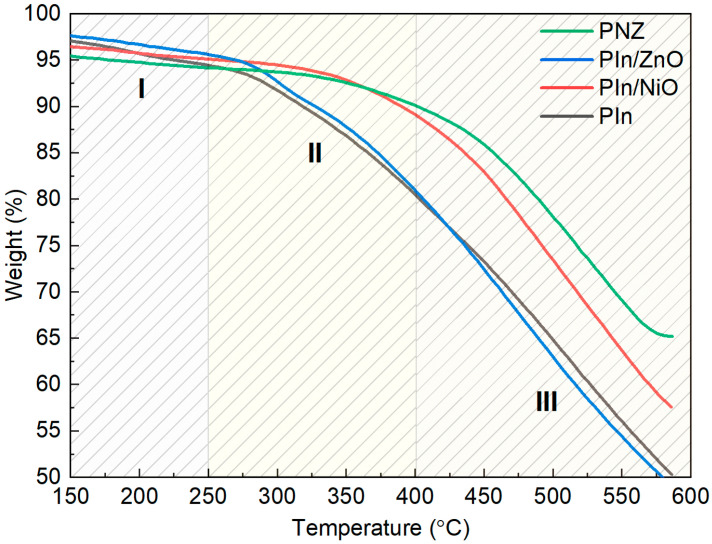
TGA spectra of the synthesized PIn and its metal composites.

The thermogravimetric analysis provides valuable information about the thermal stability of polymers for applications in technologies at elevated temperatures. At high temperatures, a loss in electrical conductivity is typically observed, which can be attributed to a de-doping of the polymer chain. The dopant loss limits the application of polymers in supercapacitors and other devices [24]. Figure 4 shows the TGA analysis of PIn, PIn/NiO, PIn/ZnO, and PNZ-3.

Each polymer and composite exhibits a three-step thermal decomposition. The first step, close to 200–250 °C with a mass loss of 3 to 4%, refers to the de-doping of DBSA. The second step is associated with eliminating oligomers with low weight loss of 5–6%. It occurs in a temperature range from 250–350 °C for PIn and PIn/ZnO. In contrast, for PIn/NiO, the upper limit reaches 365 °C, and for PNZ-3, it reaches up to 400 °C.

In the third stage, a high weight loss (26–42%) occurs at a high rate due to the polymer degradation chain at 300–600 °C for PIn and PIN/ZnO. The process starts at 355 °C for PIN/NiO and at 400 °C for PNZ-3. Compared to PIn, incorporating ZnO did not significantly change the thermal stability. In contrast, incorporating NiO improved the thermal stability of PIn/NiO and PNZ-3 to a large extent. The total weight of PIn, PIn/ZnO, and PIb/NiO after reaching 600 °C was 50.1%, 49.7%, and 57.6%, respectively. PNZ retained the highest residual mass of about 65.2%, indicating that PNZ did not decompose entirely at such high temperatures. The high thermal stability of PNZ-3 suggests that incorporating NiO and ZnO in combination increases thermal stability [22,24,27,32,33].

In addition, Figure S5 shows a comparative TGA diagram of PNZ-1 to -4 composites. It illustrates that the stability of PNZ increases with increasing ZnO concentration up to PNZ-3. Further increase leads to a decrease in thermal stability. These findings agree with previous literature reports [32]. The TGA results suggest that using a thermally resistant PNZ-3 composite could be beneficial for devices operating at high temperatures [32].

### 3.2. Electrochemical Characterisation of the Polyindole Composites

**Figure 5:**
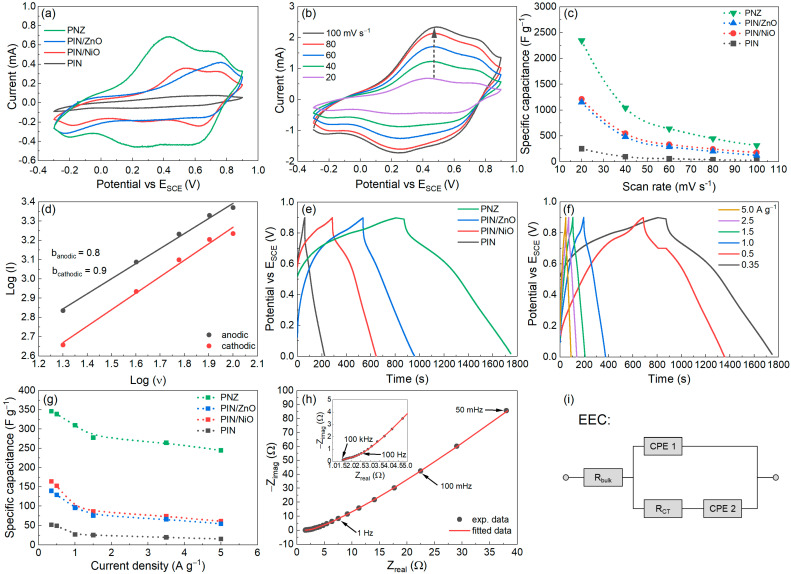
(**a**) CVs of PIn, PIn/NiO, PIn/ZnO, and PNZ-3 in three-electrode setup at 20 mV s^−1^ in a potential range of 0 V to 0.9 V, (**b**) CVs of PNZ-3 at different scan rates, (**c**) calculated specific capacitances at different scan rates (20–100 mV s^−1^) from the CVs of PNZ-3, (**d**) logarithmic correlation of the current response of PNZ-3 to the scan rate, (**e**) comparison of the GCD profiles of PIn, PIn/NiO, PIn/ZnO, and PNZ-3 at a current density of 1 A g^−1^ in the potential range of 0 V to 0.9 V, (**f**) GCD curve of PNZ at different current densities (0.35 to 5.0 A g^−1^ in a potential range of 0 V to 0.9 V), (**g**) specific capacitances of PIn, PIn/NiO, PIn/ZnO, and PNZ at different current densities (0.35 to 5.0 A g^−1^), (**h**) EIS of PNZ at V_DC_ = 200 mV in the frequency range of 100 kHz to 50 mHz, (**i**) electrical equivalent circuit (EEC) used for fitting the EIS spectra (the mass of polymer was 0.2 mg for all fabricated electrodes, and all CV, GCD, and EIS plots were recorded in 1.0 M H_2_SO_4_ aqueous solution).

To determine their electrochemical properties and performance as supercapacitor electrode materials, the prepared PIn and its metal oxide composites were studied by CV, GCD, and EIS in a half-cell setup (Figure 5).

The cyclic voltammograms of the four materials were measured in a potential range from −0.3 V to 0.9 V with a scan rate of 20 mV s^−1^ in an aqueous 1.0 M H_2_SO_4_ solution (Figure 5a). The PIn shows a widely spread redox transition with a low maximum current within the measurement window. In addition, a slight irreversibility between the forward and reverse reactions can be seen. A high internal resistance may explain the weak redox properties in the active material [24]. In contrast to the pure PIn, all metal oxide composites show quasi-reversible redox couples, which occur due to doping and de-doping processes during the redox reactions of the polymer chain. The first redox transition is related to the one-electron redox reaction of the PIn chain. In the second redox transition, a proton-coupled electron transfer of the polymer occurs. Due to the different structures of PIn/NiO, PIn/ZnO, and PNZ-3, the onset of the redox reactions varies strongly. PIn/ZnO differs from the other composites in that the redox transitions are at high potentials of ~0.52 V and ~0.78 V vs. SCE. PIn/NiO and PNZ-3 possess similar redox behavior, with two characteristic redox pairs.

The first redox transition for PIn/NiO is at ~0.41 V, while that of PNZ occurs at ~0.31 V, and the second redox transition is at ~0.72 V for both. PNZ has the most significant absolute currents of all the electrode materials—this means that the best pseudocapacitive properties are obtained for the ternary composite [3,24].

Each electrode material’s electrochemical charge storage capability was compared by determining its specific capacitances. CVs were measured at different scan rates from 20 to 100 mV s^−1^ and determined using Equation (1) (Figure 5b,c). With increasing scan rates, the typical increase in absolute currents is observed. For PNZ-3, the peak potential position shifts only slightly in the direction of the scan. The effect indicates good mass transport inside the porous composite electrode [3,24].

The highest specific capacitance of 2345.8 F g^−1^ was obtained for the PNZ ternary hybrid electrode at 20 mV s^−1^. This trend is not surprising, since a greater portion inside the composite is utilized for the redox reaction at low scan rates. Less specific capacitances of pure PIn, PIn/NiO, and PIn/ZnO were calculated to be 251.1 F g^−1^, 1210.4 F g^−1^, and 1149.7 F g^−1^, respectively, at the same scan rate. Thus, the improved electrochemical charge storage of the PNZ-3 composite electrode is attributed directly to the doping with NiO and ZnO.

As the scan rate increases, an exponential trend in the decrease of specific capacitance is observed for all active materials. This trend emerges because the time for the penetration of electrolyte ions into the porous active materials is lower, and mass transport limitations occur more strongly. Consequently, the charge storage is lower [34,35,36,37].

To gain a deeper understanding of the charge storage mechanism of the processes at the electrode–electrolyte interface, the dependence of the absolute current with the scan rate was analyzed according to Equation (8) [22].

(8) log(I)=log(a)+b log(ν)

The value for *b* gives information about the charge storage mechanism (Figure 5c). For example, suppose *b* = 0.5; the charge storage process is diffusion-controlled, whereas a surface-controlled process prevails for *b* = 1.0. Typical for active materials in supercapacitor electrodes is a value of 0.7 to 1.0. For PNZ-3, a value of 0.8 was determined for oxidation and 0.9 for reduction. The value suggests that the charge storage process is surface-dominated, but there is still an influence of diffusion-controlled mass transport on the performance [22,24].

Figure 5e shows the GCD profiles of PIn and the metal oxide composites at a current density of 1.0 A g^−1^. The asymmetric and distorted triangular profiles in the potential range from 0 V to 0.9 V originate from the pseudocapacitive behavior [22,38]. The shortest charge and discharge times were observed for PIn, followed by PIn/NiO, then PIn/ZnO, and the longest times were observed for PNZ-3. Figure 5f shows the current density dependence on the charge and discharge behavior of PNZ-3. Lower charge/discharge times are observed for higher current densities. They result in lower specific capacitance according to Equation (2). For PNZ-3, the highest specific capacitance of 346.1 F g^−1^ is obtained at 0.35 A g^−1^, which is much larger than that of PIn/NiO (163.9 F g^−1^), PIn/ZnO (139.6 F g^−1^), and PIn (51.9 F g^−1^) at the same current densities. As the current density increased, the specific capacitance decreased for all the fabricated electrodes [22,24,27]. It is interesting to note that above the current density of 1.5 A g^−1^, the decrease in capacitance takes on a linear trend. This results in the fact that at a high current density of 5.0 A g^−1^, an excellent specific capacitance of 244.4 F g^−1^ is still obtained. The reason for this is the low diffusion dependence, which has already been shown by CV analysis [39].

For an in-depth understanding of the electrochemical processes of the PNZ-3 composite, an electrochemical impedance spectroscopic analysis was performed. The EIS spectrum was measured in a frequency range between 100 kHz and 50 mHz. The Nyquist plot is shown in Figure 5g. The spectrum can be divided into three regions: (1) the intercept with the *x*-axis describes the sum of all ohmic resistances (R_Bulk_) of the electrode, (2) in the range between 90 kHz and 1 Hz, all processes involved in the electrochemical reactions occur (R_CT_, CPE1), and (3) the frequency range lower than 1 Hz results from limiting transport processes (CPE2) [21,39]. The electrical equivalent circuit shown in Figure 5i was used to determine the individual contributions to the total spectrum. A value of 2.95 Ω was obtained for the ohmic resistance R_Bulk_, indicating the excellent electrical conductivity of the PNZ-3 composite and low contact resistance with the gold current collector and the electrolyte. A value of 61.3 Ω was determined for the charge transfer resistance R_CT_. The low R_CT_ indicates rapid redox reversibility at the electrode–electrolyte interface. Moreover, CPE1 (Q^n^ = 1.04e^−3^ Ss^n^, n = 0.6) expresses the pseudocapacitive nature of the PNZ hybrid electrode, which is typical of an energy storage mechanism. CPE2 (Q^n^ = 108.5e^−6^ Ss^n^, n = 0.7) describes a non-optimal Warburg diffusion. The value for n > 0.5 indicates that the influence of diffusion on the charge storage mechanism is moderate [22,24].

In summary, the CV, GCD, and EIS analyses show that the best capacitive performance is obtained for the PNZ-3 electrode, making it the most suitable for supercapacitors.

### 3.3. Analysis of the PNZ Composite as Faradaic Supercapacitor

**Figure 6:**
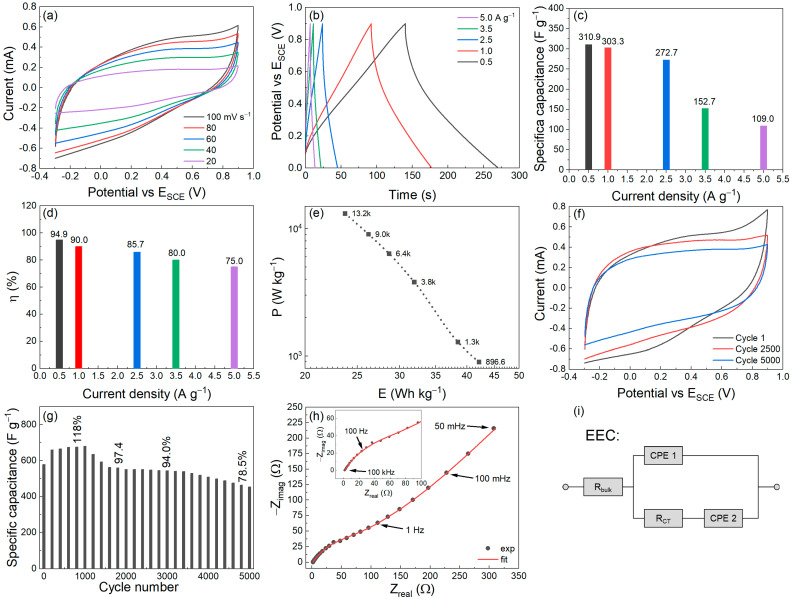
(**a**) CVs of PNZ in two-electrode setup at different scan rates (20 to 100 mV s^−1^) in a potential range between -0.3 V and 0.9 V, (**b**) GCD of PNZ at different current densities (0.5 to 5.0 A g^−1^) in the same potential range, (**c**) calculated specific capacitances of PNZ at the different current densities, (**d**) coulombic efficiencies of PNZ at the different current densities, (**e**) Ragoné plot of PNZ, (**f**) CVs of PNZ at a scan rate of 100 mV s^−1^ after aging in a potential range between -0.3 V and 0.9 V, (**g**) cycle stability of PNZ at 100 mV s^−1^ over 5000 cycles, (**h**) EIS spectra of PNZ in two-electrode setup at V_DC_ = 200 mV in the frequency range of 100 kHz to 50 mHz, (**i**) electrical equivalent circuit used for EIS fitting. (The mass of polymer was 0.2 mg for all fabricated electrodes, and all CV, GCD, and EIS plots were recorded in 1.0 M H_2_SO_4_ aqueous solution).

For realistic performance analysis of the PNZ composite, it was assembled in a symmetrical supercapacitor and investigated using CV, GCD, and EIS in a two-electrode setup. Figure 6a shows the CV curves of the PNZ supercapacitor at different scan rates ranging from 20 to 100 mV s^−1^. The symmetrical setup results in obtaining distorted, nearly rectangular voltammograms. The current response to the scan rate is quasi-linear. Figure 6b shows the GCD waveforms of the supercapacitor at different current densities. The triangular charge and discharge waveforms are almost identical. This behavior is found in supercapacitors with fast redox properties and capacitance properties. Equation (3) can be used to calculate the specific capacitance of the symmetrical supercapacitor. A high specific capacitance of 310.9 F g^−1^ at 0.5 A g^−1^ was obtained. Increasing the current density up to 2.5 A g^−1^ resulted in only a tiny decrease in capacitance to 272.7 F g^−1^. Further increase in current density resulted in a considerable loss of capacitance. A possible reason is the slight existing kinetic differences in the oxidation and reduction processes [22,40].

Figure 6d shows the coulombic efficiency. It indicates the efficiency of the charge-to-discharge energy ratio and is a measure of the energy efficiency of the supercapacitor.

The symmetrical PNZ supercapacitor shows an excellent coulombic efficiency of 94.9% at 0.5 A g^−1^, indicating good charge transfer [22]. At higher current densities, the efficiency does not decrease moderately but is still in a range that is very suitable for supercapacitor applications.

A Ragone diagram is used to understand the relationship between energy density and power density (Figure 6e). A high energy density of 42.1 Wh kg^−1^ is achieved with a reasonable power density of 0.89 kW kg^−1^. Furthermore, the device exhibited an energy density of 23.7 Wh kg^−1^ with a power density of 13.2 kW kg^−1^. The results suggest that the PNZ supercapacitor is best used for high-power density applications at an intermediate energy density [22,24,41].

Furthermore, the cycling stability of the supercapacitor was investigated at a scan rate of 100 mV s^−1^ for 5000 consecutive CV cycles in 1 M H_2_SO_4_ in the potential range of -0.3 to 0.9 V. From Figure 6f, it can be seen that the maximum current decreases between the first, 2500th, and 5000th cycle. This decrease corresponds to the loss in charge storage and specific capacitance. The capacitance curve shows an exciting trend in Figure 6g. The specific capacitance of the symmetric supercapacitor initially increases (up to 1000 cycles) and then decreases. The initial increase in specific capacitance is due to the structural change during the redox processes, which most likely leads to an increase in the active surface area. After 5000 CV cycles, about 78.5% of the initial specific capacity remains. From these results, it can be concluded that the fabricated PNZ-3 composite is a promising electrode material for practical use in high-performance supercapacitors.

For comparison with other polyindole electrode materials, various hybrid capacitors are shown in Table 1.

Similar to the three-electrode arrangement, an EIS measurement of the PNZ supercapacitor was performed in the frequency range between 100 kHz and 50 mHz. The Nyquist plot is shown in Figure 6h. The bulk resistance in the PNZ supercapacitor is 1.4 Ω. A small open semicircle for charge penetration can be seen. The charge transfer resistance R_CT_ is 73.6 Ω, and a value of Q^n^ = 0.48e^−3^ Ss^n^ and n = 0.8 was obtained for the associated CPE1 element. The values imply good charge transport and a significant capacitive contribution of the surface-controlled processes to the performance. For the CPE2 element, a value of Q^n^ = 1.30e^−6^ Ss^n^ and n = 0.67 was obtained. This shows that in the symmetric supercapacitor, the influence of the diffusion-involved processes is more vital than in the three-electrode setup [22,43]. The more significant influence of diffusion is consistent with the discharge capacitances of the GCD measurements; with higher current densities, the slower transport leads to capacitance losses.

## 4. Conclusions

A direct one-pot chemical synthesis of PIn and its NiO- and ZnO-containing binary and ternary composites was reported. Different compounds with different ZnO concentrations were prepared for the ternary PIn/NiO/ZnO composite. Only the composite with a ratio of 1:1.4 equivalents of NiO:ZnO (PNZ-3) showed good performance and was further investigated. PIn and its metal oxide composites possessed a porous structure with a large surface area, and PNZ exhibited the most homogeneous structure. Using EDX, FTIR, and XRD, the successful incorporation of NiO and ZnO into the polymer structure was demonstrated. The incorporation of the metal oxides is directly related to their electrochemical performance. In particular, the combination of PIn with both metal oxides in PNZ-3 showed excellent charging and discharging properties. Excellent capacities, capacitance retention, and cycling stability were obtained for the PNZ electrodes. Performance analysis of the symmetrical PNZ supercapacitor showed that it is best suited for high-power-density applications at average energy densities of 10 to 30 Wh kg^−1^. The property of high power density could be confirmed by employing EIS. The PNZ capacitor has very efficient and fast charge transfer and low diffusive transport limitations. The results show that ternary polyindoles are promising electrode materials for energy storage applications. They are easy and direct to prepare and are chemically and electrochemically robust and efficient.

## Figures and Tables

**Table 1:** Comparison of various polyindole metal oxide supercapacitors.

Electrode Material	E (Wh kg^−1^)	P (kW kg^−1^)	Stability/Cycles ^a^	Ref.
CNTs/PIn/Co_3_O_4_	42.9	0.4	90.2%/5000 (1 A g^−1^)	[10]
PIn/CB/MoS_2_	8.7	2.03	92.3%/5000 (1 A g^−1^)	[12]
V_2_O_5_/PIn@AC	38.7	0.9	91.1%/5000 (1 A g^−1^)	[13]
PIn/NiO	17.5	19.3	114%/10,000 (1 A g^−1^)	[22]
PIn/Nd_2_O_3_	8.9	1.0	97.0%/5000 (3 A g^−1^)	[42]
PIn/Gd_2_O_3_	<8.9	1.0	85.0%/5000 (3 A g^−1^)	[43]
PNZ	42.1	13.7	78.5%/5000 (100 mV s^−1^)	this work

^a^: all experiments were performed in acidic aqueous electrolyte.

## Data Availability

The data presented in this study are available upon request from the corresponding author.

## Supplementary Materials

The following supporting information can be downloaded at https://www.mdpi.com/article/10.3390/nano13030618/s1. Figure S1: SEM images of the PNZ composites with varying ZnO concentration; Figure S2: EDX spectra and elemental mapping analysis of (a) PIn, (b) PIn/NiO, (c) PIn/ZnO, and (d) PNZ-3; Figure S3: EDX spectra and elemental mapping analysis of PNZ ternary composites prepared with varying content of ZnO; Figure S4: FTIR spectra of NiO, ZnO, and PNZ ternary composites; Figure S5: XRD spectra of pure NiO, pure ZnO, and PNZ ternary composites; Figure S6: TGA of PNZ ternary composites synthesized with different concentrations of ZnO.

## References

[1] Kubra K.T., Javaid A., Patil B., Sharif R., Salman A., Shahzadi S., Siddique S., Ghani S. (2019). Synthesis and characterization of novel Pr_6_O_11_/Mn_3_O_4_ nanocomposites for electrochemical supercapacitors. Ceram. Int..

[2] Zhao X., Mao L., Cheng Q., Li J., Liao F., Yang G., Xie L., Zhao C., Chen L. (2020). Two-dimensional spinel structured Co-based materials for high performance supercapacitors: A Critical Review. Chem. Eng. J..

[3] Huang C., Hao C., Zheng W., Zhou S., Yang L., Wang X., Zhu L. (2020). Synthesis of polyaniline/nickel ox-ide/sulfonated graphene ternary composite for all-solid-state asymmetric supercapacitor. Appl. Surf. Sci..

[4] Gao P., Chen Z., Gong Y., Zhang R., Liu H., Tang P., Chen X., Passerini S., Liu J. (2020). The role of cation vacancies in electrode materials for enhanced electrochemical energy storage: Synthesis, advanced characterization, and fundamentals. Adv. Energy Mater..

[5] Ramesan M.T., Anjitha T., Parvathi K., Anilkumar T., Mathew G. (2018). Nano zinc ferrite filler incorporated polyindole/poly(vinyl alcohol) blend: Preparation, characterization, and investigation of electrical properties. Adv. Polym. Technol..

[6] Yu M., Qiu W., Wang F., Zhai T., Fang P., Lu X., Tong Y. (2015). Three dimensional architectures: Design, assembly and application in electrochemical capacitors. J. Mater. Chem. A.

[7] Isa N.M., Baharin R., Majid R.A., Rahman W.A.W.A. (2017). Optical properties of conjugated polymer: Review of its change mechanism for ionizing radiation sensor. Polym. Adv. Technol..

[8] Milan B.P., Changho Y., Han J.K. (2020). Synthesis of conducting bifunctional polyaniline@Mn-TiO_2_ nanocomposites for supercapacitor electrode and visible light driven photocatalyst. Catalysts.

[9] Yanik M.O., Yigit E.A., Akansu Y.E., Sahmetlioglu E. (2017). Magnetic conductive polymer-graphene nanocomposites based supercapacitors for energy storage. Energy..

[10] Zhou Q., Zhu D., Ma X., Xu J., Zhou W., Zhao F. (2016). High-performance capacitive behavior of layered reduced graphene oxide and polyindole nanocomposite materials. RSC Adv..

[11] Ram B.C., Sarfaraz A. (2022). Mesoporous complexion and multi-channeled charge storage action of PIn-rGO-TiO_2_ ternary hybrid materials for supercapacitor applications. J. Energy Storage.

[12] Majumder M., Choudhary R.B., Koiry S.P., Thakur A.K., Kumar U. (2017). Gravimetric and volumetric capacitive performance of polyindole/carbon black/MoS_2_ hybrid electrode material for supercapacitor applications. Electrochim. Acta.

[13] Zhou X., Chen Q., Wang A., Xu J., Wu S., Shen J. (2016). The bamboo-like composites of V_2_O_5_/polyindole and activated carbon cloth as electrodes for all-solid-state flexible asymmetric supercapacitors. Appl. Mater. Interfaces.

[14] Ramesan M.T., Santhi V. (2018). Synthesis, characterization, conductivity and sensor application study of polypyrrole/silver doped nickel oxide nanocomposites. Compos. Interfaces.

[15] Xie Y., Sha X. (2018). Electrochemical cycling stability of nickel (II) coordinated polyaniline. Synth. Met..

[16] Fua L., Fub Z. (2015). Plectranthus amboinicus leaf extract assisted biosynthesis of ZnO nanoparticles and their photocatalytic activity. Ceram. Int..

[17] Thadathil A., Pradeep H., Joshy D., Ismail Y.A., Periyat P. (2022). Polyindole and polypyrrole as a sustainable platform for environmental remediation and sensor applications. Mater. Adv..

[18] Mehsenpour M., Naderi M., Arash G., Aghabararpour M., Haghshenas D.F. (2021). Fabrication of paper-based carbon/graphene/ZnO aerogel composite decorated by polyaniline nanostructure: Investigation of electrochemical properties. Ceram. Int..

[19] Rashti A., Wang B., Hassani E., Feyzbar-Khalkhali-Nejad F., Zhang X., Oh T.-S. (2020). Electrophoretic deposition of nickel cobaltite/polyaniline/rGO composite electrode for high performance all-solid-state asymmetric supercapacitors. Energy Fuels.

[20] Rajesh M., Manikandan R., Kim B.C., Becuwe M., Yu K.H., Raj C.J. (2020). Electrochemical polymerization of chloride doped PEDOT hierarchical porous nanostructure on graphite as A potential electrode for high performance supercapacitor. Electrochim. Acta.

[21] Waikar M.R., Rasal A.S., Shinde N.S., Dhas S.D., Moholkar A.V., Shirsat M.D., Chakarvarti S.K., Sonkawade R.G. (2020). Electro-chemical performance of Polyaniline based symmetrical energy storage device. Mater. Sci. Semicond. Proc..

[22] Begum B., Bilal S., Shah A.H.A., Röse P. (2022). Synthesis, characterization and electrochemical performance of a redox-responsive polybenzopyrrole @ nickel oxide nanocomposite for robust and efficient faradaic. Nanomaterials.

[23] Hossain A., Abdallah Y., Ali M.A., Masum M.M.I., Li B., Sun G., Meng Y., Wang Y., An Q. (2019). Lemon-fruit-based green synthesis of zinc oxide nanoparticles and titanium dioxide nanoparticles against soft rot bacterial pathogen dickey dadantii. Biomolecules.

[24] Begum B., Bilal S., Shah A.U.H.A., Röse P. (2021). Physical, chemical, and electrochemical properties of redox-responsive polybenzopyrrole as electrode material for faradaic energy storage. Polymers.

[25] Hajera G., Anwar-ul-Haq S., Ulrike K., Salma B. (2020). Study on direct synthesis of energy efficient multifunctional polyanilinegraphene oxide nanocomposite and its application in aqueous symmetric supercapacitor devices. Nanomaterials.

[26] Yang H., Xu H., Li M., Zhang L., Huang Y., Hu X. (2016). Assembly of NiO/Ni(OH)_2_/PEDOT nanocomposites on contra wires for fiber-shaped flexible asymmetric supercapacitors. ACS Appl. Mater. Interfaces.

[27] Batool A., Kanwal F., Imran M., Jamil T., Siddiqi S.A. (2012). Synthesis of polypyrrole/zinc oxide composites and study of their structural, thermal and electrical properties. Synth. Met..

[28] Inamuddin, Shakeel N., Ahamed M.I., Kanchi S., Kashmery H.A. (2020). Green synthesis of ZnO nanoparticles decorated on polyindole functionalized-MCNTs and used as anode material for enzymatic biofuel cell applications. Sci. Rep..

[29] Verma C.J., Keshari A.S., Dubey P., Prakash R. (2020). Polyindole modifed g-C3N4 nanohybrids via in-situ chemical polymerization for its improved electrochemical performance. Vacuum.

[30] Shyamala S., Muthuchudarkodi R.R. (2018). Polyindole based zinc oxide nanocomposite-synthesis and characterization. Syst. Evol. Microbiol..

[31] Shah A.H.A., Yasmeen N., Rahman G., Bilal S. (2017). High electrocatalytic behavior of Ni impregnated conducting polymer coated platinum and graphite electrodes for electrooxidation of methanol. Electrochim. Acta.

[32] Cai X., Cui X., Zu L., Zhang Y., Gao X., Lian H., Liu Y., Wang X. (2017). Ultra-high electrical performance of nano nickel oxide and polyaniline composite materials. Polymers.

[33] Mohsen R.J., Morsi S.M.M., Selim M.M. (2019). Electrical, thermal, morphological, and antibacterial studies of synthesized polyaniline/zinc oxide nanocomposites. Polym. Bull..

[34] Li X., Zhang C., Xin S., Yang Z., Li Y., Zhang D., Yao P. (2016). Facile synthesis of MoS_2_/reduced graphene oxide@ polyaniline for high-performance supercapacitors. ACS Appl. Mater. Interfaces.

[35] Bilal S., Begum B., Gul S., Shah A.H.A. (2018). PANI/DBSA/H_2_SO_4_; A promising and highly efficient electrode material for aqueous supercapacitors. Synth. Met..

[36] Syduly B.S., Palaniappan S., Sirnivas P. (2013). Nano fibre polyaniline containing long chain and small molecule dopants and carbon composites for supercapacitors. Electrochim. Acta.

[37] Fathi M., Saghafi M., Mahboubi F., Mohajerzadeh S. (2014). Synthesis and electrochemical investigation of polyaniline/unzipped carbon nanotube composites as electrode material in supercapacitors. Synth. Met..

[38] Zhang J., Liu Y., Guan H., Zhao Y., Zhang B. (2017). Decoration of nickel hydroxide nanoparticles onto polypyrrole nanotubes with enhanced electrochemical performance for supercapacitors. J. Alloys Compd..

[39] Zhou X., Wang A., Pan Y., Yu C., Zou Y., Zhou Y., Chen Q., Wu S. (2015). Facile synthesis of a Co3O4@carbon nanotubes/polyindole composite and its application in all-solid-state flexible supercapacitors. J. Mater. Chem. A.

[40] Shah A.-u.-H.A., Khan M.O., Bilal S., Rahman G., Hung H.V. (2018). Electrochemical co-deposition and characterization of polyaniline and manganese oxide nanofibrous composites for energy storage properties. Adv. Polym. Technol.

[41] Shah S.S., Das H.T., Barai H.R., Aziz M. (2022). Boosting the electrochemical performance of polyaniline by one-step electrochemical deposition on nickel foam for high-performance asymmetric supercapacitor. Polymers.

[42] Majumder M., Choudhary R.B., Thakur A.K., Rout C.S., Gupta G. (2018). Rare earth metal oxide (RE_2_O_3_; RE = Nd, Gd, and Yb) incorporated polyindole composites: Gravimetric and volumetric capacitive performance for supercapacitor applications. New J. Chem..

[43] Singu B.S., Palaniappan S., Yoon K.R. (2016). Polyaniline-nickel oxide nanocomposite for supercapacitor. J. Appl. Electrochem..

