# Supplementary material for: Polyindole Embedded Nickel/Zinc Oxide Nanocomposites for High-Performance Energy Storage Applications
Source: Nanomaterials (Basel). 2023 Feb 3;13(3):618. doi: 10.3390/nano13030618 (PMC9921157; doi:10.3390/nano13030618)
Supplement: Supplementary file 1 [file nanomaterials-13-00618-s001.zip › nanomaterials-2195554-supplementary.pdf]

nanomaterials

MDPI

Supplementary Materials

Polyindole Embedded Nickel/Zinc Oxide Nanocomposites for High-Performance Energy Storage Applications

Huriya Humayun [1], Bushra Begum [1], Salma Bilal [1,*], Anwar ul Haq Ali Shah [2] and Philipp Röse [3,*]

[1] National Centre of Excellence in Physical Chemistry 1, University of Peshawar, Peshawar 25120, Pakistan
[2] Institute of Chemical Science, University of Peshawar, Peshawar 25120, Pakistan
[3] Institute for Applied Materials—Electrochemical Technologies, Karlsruhe Institute of Technology (KIT), 76131 Karlsruhe, Germany

Table of Content	Page No.
**SEM Analysis**	**2**
**EDX Analysis**	**3**
**FTIR Analysis**	**5**
**XRD Analysis**	**6**
**TGA Analysis**	**7**
**Reference**	**7**

1. SEM images of PNZ-1, PNZ-2, PNZ-3 and PNZ-4

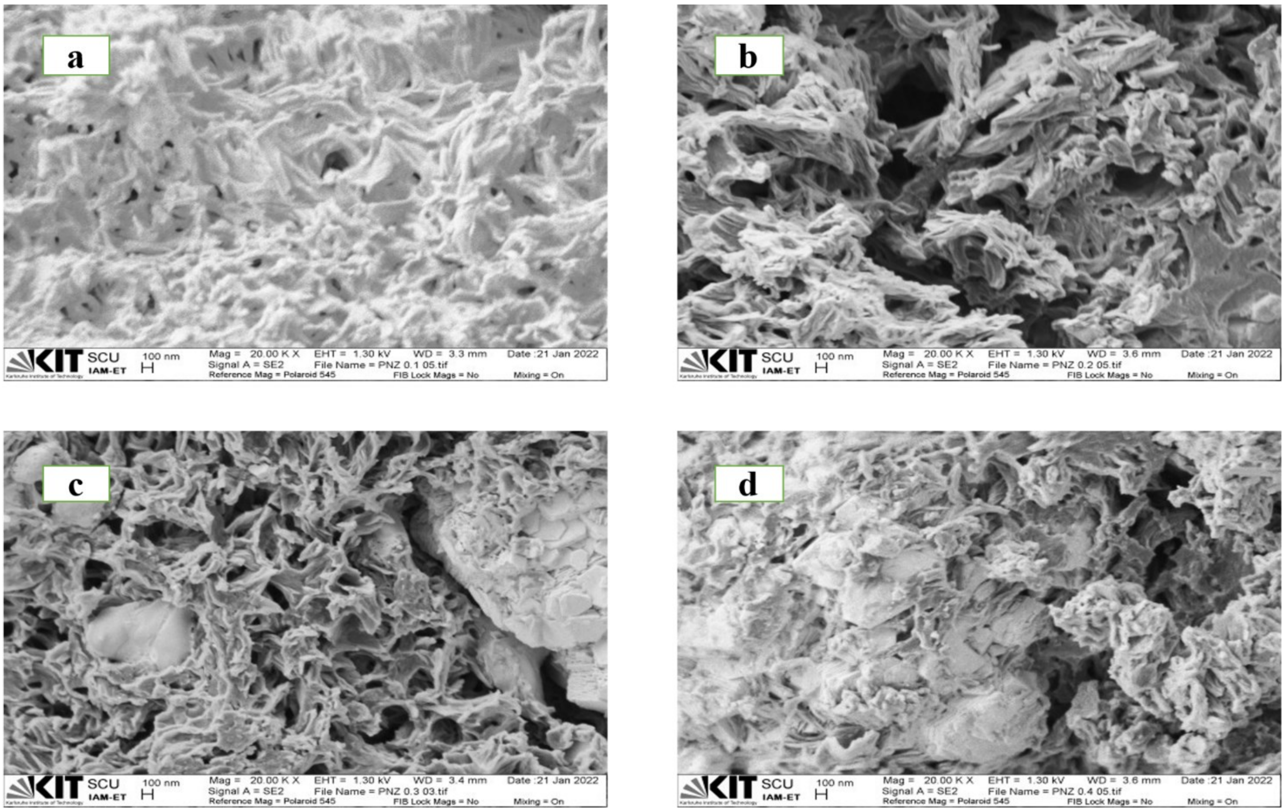

Figure S1. SEM images of the PNZ composites with varying ZnO concentration: (a) PNZ-1, (b) PNZ-2, (c) PNZ-3, and (d) PNZ-4.

The SEM image of PNZ-1 shows intertwined nanostructures with very few pores (Figure S1a). When the ZnO content was increased to 0.2 g (Figure S1b), the intertwined nanostructures became more uniform and showed some particles on the surface. The pores are extensive. The interconnected uniform nanofibers appeared when the amount of ZnO was further increased (0.3 g). In addition, some large agglomerated particles and micropores can also be seen in Figure S1c. The surface morphology of PNZ-0.4 becomes more heterogeneous and shows agglomerated macro- and microparticles with vacancies of varying size (Figure S1d). Thus, the influence of ZnO on the surface structure of the ternary PNZ composite is powerful.

2. EDX of PIn and its composites

Furthermore, the effect of ZnO concentration on the EDX spectra is demonstrated in Figure S2 and S3.

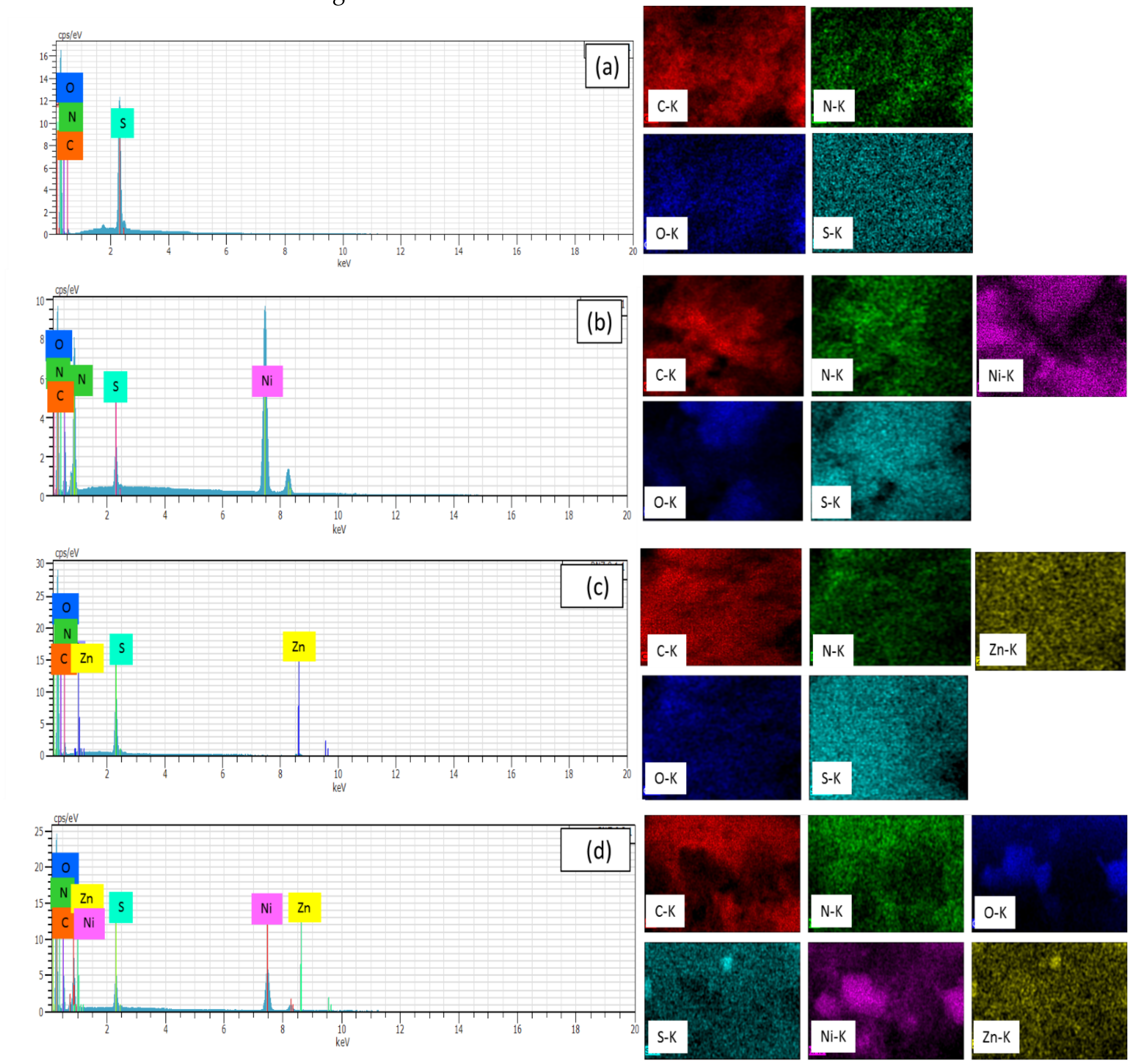

Figure S2. EDX spectra and elemental mapping analysis of (a) PIn, (b) PIn/NiO, (c) PIn/ZnO, and (d) PNZ-3.

Figure S3 shows the PNZ-composites with varying ZnO content. The increase in ZnO amount during the polymerization, the increase in weight % of Zn occurred from 0.64% in PNZ-0.1 to 5.88% in PNZ-0.4 ternary composite.

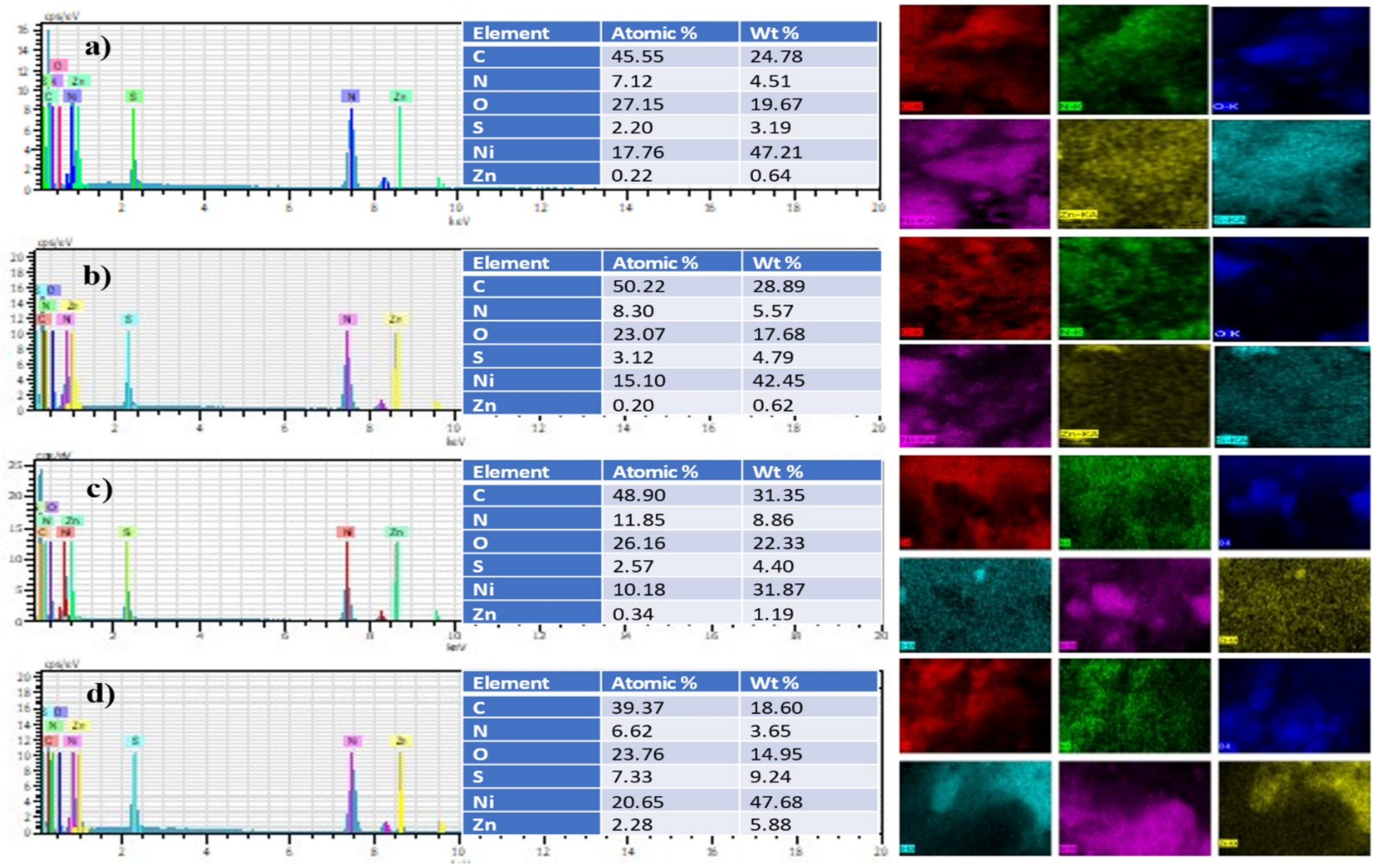

a)

Element	Atomic %	Wt %
C	45.55	24.78
N	7.12	4.51
O	27.15	19.67
S	2.20	3.19
Ni	17.76	47.21
Zn	0.22	0.64

b)

Element	Atomic %	Wt %
C	50.22	28.89
N	8.30	5.57
O	23.07	17.68
S	3.12	4.79
Ni	15.10	42.45
Zn	0.20	0.62

c)

Element	Atomic %	Wt %
C	48.90	31.35
N	11.85	8.86
O	26.16	22.33
S	2.57	4.40
Ni	10.18	31.87
Zn	0.34	1.19

d)

Element	Atomic %	Wt %
C	39.37	18.60
N	6.62	3.65
O	23.76	14.95
S	7.33	9.24
Ni	20.65	47.68
Zn	2.28	5.88

Figure S3. EDX spectra and elemental mapping analysis of PNZ ternary composites prepared with varying content of ZnO (a) PNZ-0.1, (b) PNZ-0.2 (c) PNZ-0.3, and (d) PNZ-0.4.

3. FTIR Analysis

Figure S3 presents FTIR spectra of pure NiO, pure ZnO, and PNZ ternary hybrids prepared with varying amounts of ZnO. The FTIR spectra of NiO and ZnO revealed characteristic peaks in the region of 613 and 507 cm^{-1}, respectively. Upon varying ZnO loading, all the PNZ ternary hybrids demonstrated nearly similar FTIR spectra with little change in peak positions and their intensities. Therefore, the FTIR spectra confirm the association of ZnO, variation in the degree of conjugation, and sensitivity of molecular structure of the well-integrated PNZ ternary hybrids.

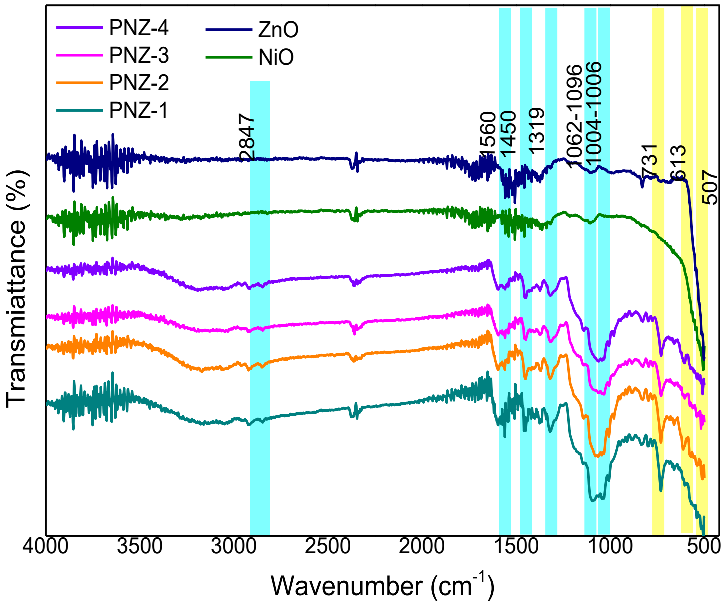

Figure S4. FTIR spectra of NiO, ZnO, and PNZ ternary composites.

4. XRD Analysis

Figure S4 illustrates the XRD patterns of NiO, ZnO, and various PNZ hybrids. The XRD pattern of NiO shows sharp characteristic peaks at 2θ=37.4°, 43.4°, 62.8°, 75.3°, and 79.2° which manifested the face-centered core crystalline structure. The diffraction peaks of NiO matches well with literature (JCPDS no. 040835). The 2θ pattern of ZnO exhibits the reflection peaks at 31.9°, 34.5°, 36.5°, 47.7°, 56.7°, 63.0°, 68.0°, and 69.2°. These characteristic lines correspond to the wurtzite structure of ZnO (JCPDS no. 36-1451) [1,2].

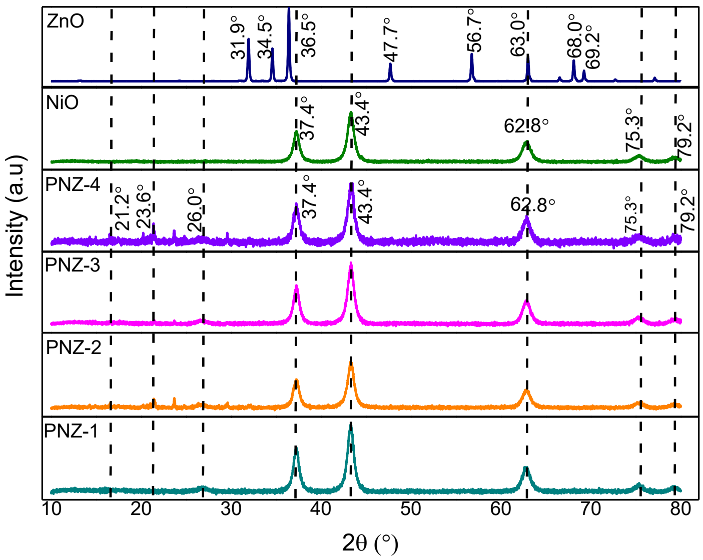

Figure S5. XRD spectra of pure NiO, pure ZnO, and PNZ ternary composites.

Additionally, the XRD spectra of all PNZ hybrids exhibited almost all diffraction patterns as found in their individual components with little variation in the peak's intensity and sharpness due to variation in synthesis parameter. The ternary composite with 0.3 g amount of ZnO shows highest peaks height that signifies the uniformly and compact arrangement of the molecules that allow the transportation of ions.

5. TGA Analysis

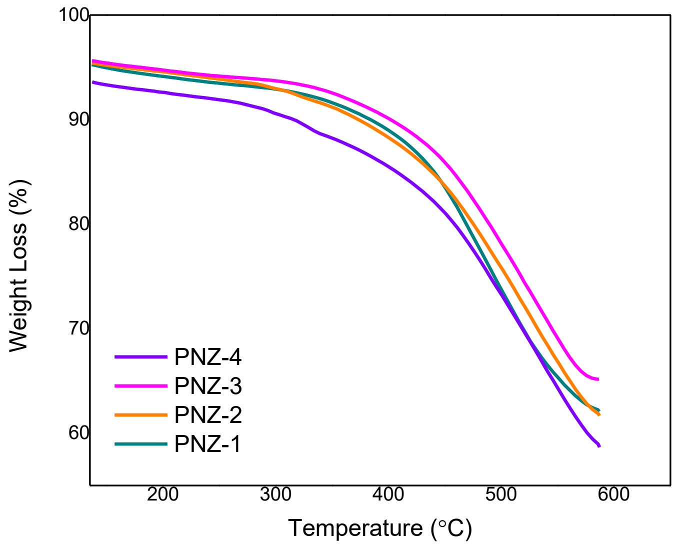

Figure S6. TGA of PNZ ternary composites synthesized with different concentrations of ZnO.

References

1. Inamuddin, Shakeel, N.; Ahamed, M. I.; Kanchi, S.; Kashmery, H. A. Green synthesis of ZnO nanoparticles decorated on polyindole functionalized-MCNTs and used as anode material for enzymatic biofuel cell applications. *Sci. Rep.* **2020**, *10*, 5052–5062.

2. Afsana, H.; Yasmine, A.; Md. A. A.; Md. M. I. M.; Bin, L.; Guochang, S.; Youqing, M.; Yanli, W.; Qianli, A., Lemon-fruit-based green synthesis of zinc oxide nanoparticles and titanium dioxide nanoparticles against soft rot bacterial pathogen dickey dadantii, Biomolecules, **2019**, 9, 863.
